# Nitroxides as Building Blocks for Nanoantioxidants

**DOI:** 10.1021/acsami.1c06674

**Published:** 2021-06-22

**Authors:** Damiano Genovese, Andrea Baschieri, Danilo Vona, Ruxandra Elena Baboi, Fabio Mollica, Luca Prodi, Riccardo Amorati, Nelsi Zaccheroni

**Affiliations:** † Department of Chemistry “Giacomo Ciamician”, University of Bologna, via Selmi 2, 40126 Bologna, Italy; ‡ Department of Chemistry “Giacomo Ciamician”, University of Bologna, via San Giacomo 11, 40126 Bologna, Italy; § Istituto per la Sintesi Organica e la Fotoreattività (ISOF), Consiglio Nazionale delle Ricerche (CNR), via Gobetti 101, 40129 Bologna, Italy; ∥ Department of Chemistry, University of Bari, via Orabona 4, I-70126 Bari, Italy

**Keywords:** nanoparticles, antioxidant, nitroxides, proton-coupled electron transfer, peroxyl radicals, lipid peroxidation

## Abstract

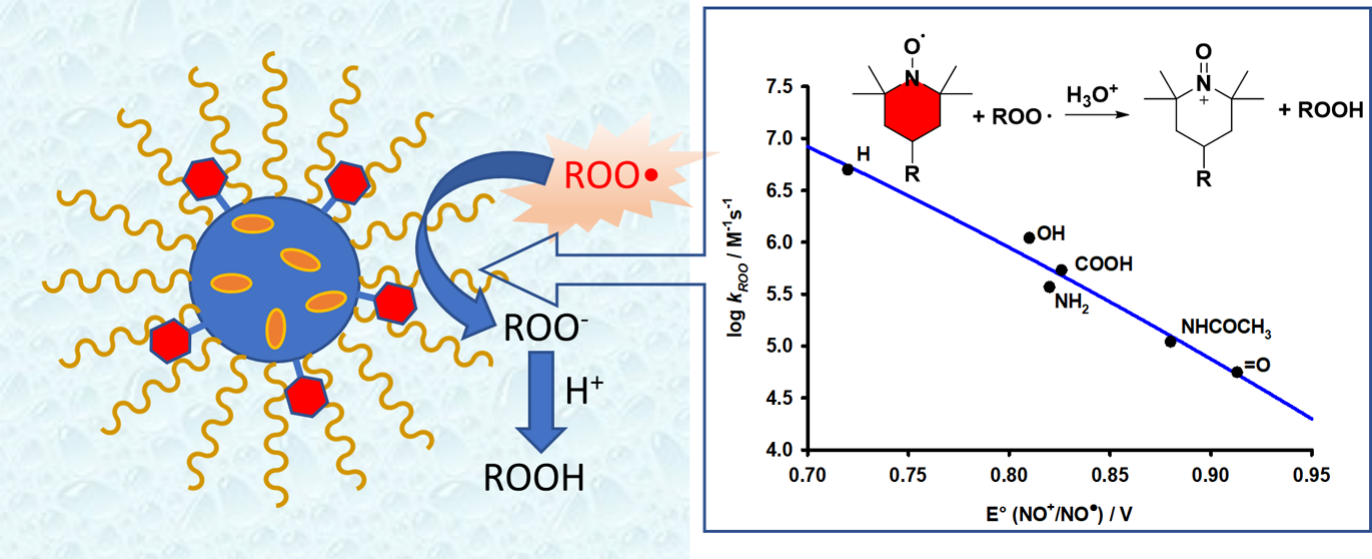

Nitroxides are an important class of radical trapping antioxidants whose promising biological activities are connected to their ability to scavenge peroxyl (ROO^•^) radicals. We have measured the rate constants of the reaction with ROO^•^ (*k*_inh_) for a series of 2,2,6,6-tetramethyl-1-piperidinyloxy (TEMPO) derivatives as 5.1 × 10^6^, 1.1 × 10^6^, 5.4 × 10^5^, 3.7 × 10^5^, 1.1 × 10^5^, 1.9 × 10^5^, and 5.6 × 10^4^ M^–1^ s^–1^ for −H, −OH, −NH_2_, −COOH, −NHCOCH_3_, −CONH(CH_2_)_3_CH_3_, and =O substituents in the 4 position, with a good Marcus relationship between log (*k*_inh_) and *E*° for the R_2_NO^•^/R_2_NO^+^ couple. Newly synthesized Pluronic-silica nanoparticles (PluS) having nitroxide moieties covalently bound to the silica surface (PluS–NO) through a TEMPO–CONH–R link and coumarin dyes embedded in the silica core, has *k*_inh_ = 1.5 × 10^5^ M^–1^ s^–1^. Each PluS-bound nitroxide displays an inhibition duration nearly double that of a structurally related "free" nitroxide. As each PluS–NO particle bears an average of 30 nitroxide units, this yields an overall ≈60-fold larger inhibition of the PluS–NO nanoantioxidant compared to the molecular analogue. The implications of these results for the development of novel nanoantioxidants based on nitroxide derivatives are discussed, such as the choice of the best linkage group and the importance of the regeneration cycle in determining the duration of inhibition.

## 1 Introduction

**Figure 1:**
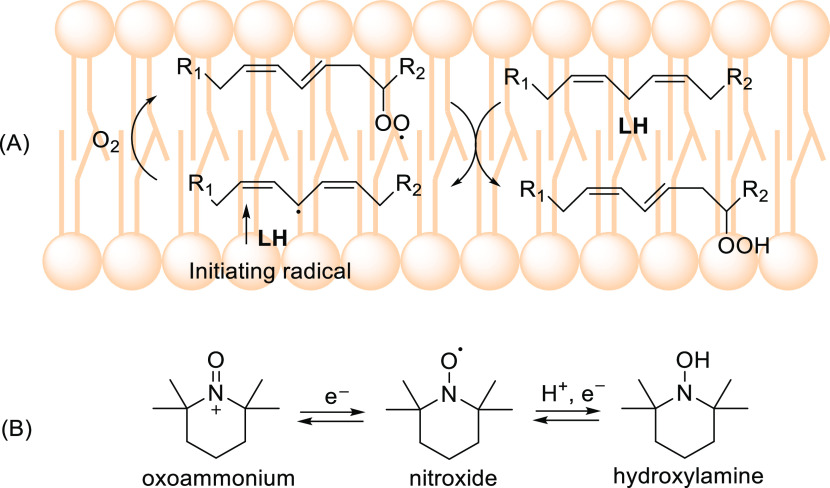
(A) Role of alkyperoxyl radicals in lipid peroxidation (LH = lipid). (B) Structure and redox states of nitroxides.

Nanomaterials with antioxidant properties (nanoantioxidants) represent an emerging strategy to counteract oxidative spoilage of organic materials and to modulate redox reactions in biological systems (see for instance lipid peroxidation in Figure 1A).^1^ They can provide large local concentration,^2^ stabilization, and controlled release of labile antioxidants,^3^ and the possibility to target specific cells or organs.^4^ The antioxidant activity can be displayed by intrinsically redox-active nanomaterials (i.e., metal oxides, melanins, lignins)^1^ or can be obtained by anchoring small-molecule antioxidants to the surface of inert scaffolds.^1^ Surface functionalization is typically performed by exploiting natural and synthetic antioxidants including glutathione,^5^ carotenoids,^6^ gallic acid,^7^ curcumin,^8^ α-tocopherol analogues,^9,10^ and butylated hydroxytoluene (BHT).^11^ Although phenols represent the most common surface-active antioxidant agents, their efficacy is drastically diminished by their instability under air.^12,13^ In water, phenols typically degrade by the deprotonation of ArOH groups, followed by the reaction with O_2_ generating superoxide (O_2_^•–^/HOO^•^) and phenoxyl radicals.^12^ In the context of our ongoing research in the field of nanoantioxidants, we envisaged that these shortcomings could be overcome using hindered nitroxides as surface-bound antioxidants.

These compounds (see Figure 1B) are a class of persistent radicals characterized by high stability in water under air.^14^ The most popular nitroxides are those belonging to the 2,2,6,6-tetramethyl-1-piperidinyloxy (TEMPO) family. Nitroxides are excellent traps for alkyl (R^•^) and alkylperoxyl radicals (ROO^•^) that are the main radicals involved in the peroxidation of organic compounds (see Figure 1A).^15−21^ Nitroxides have promising pharmacological activity such as the inhibition of ferroptosis,^22^ reduction of inflammation caused by *Mycobacterium tuberculosis*,^23^ and protection from retinopathy^24^ and from ischemia–reperfusion.^25^ Given these premises, it was surprising to find that nitroxides have received only little attention in the field of nanoantioxidants, despite the fact that there are many examples of surface-anchored nitroxides for different applications (i.e., oxidation catalysis,^26^ organic batteries,^27^ etc.). The examples that appeared so far in literature are micellar assemblies of nitroxide-poly(ethylene glycol) (PEG) surfactants,^4,28^ self-assembled amphiphilic block copolymers having nitroxide pendants,^29^ and Au-PEG-nitroxide core/shell nanoparticles.^30^ Recently biosilica extracted from microalgae has been functionalized with a TEMPO-derived radical and used as a substrate for model bone cell growth.^31^

**Figure 2:**
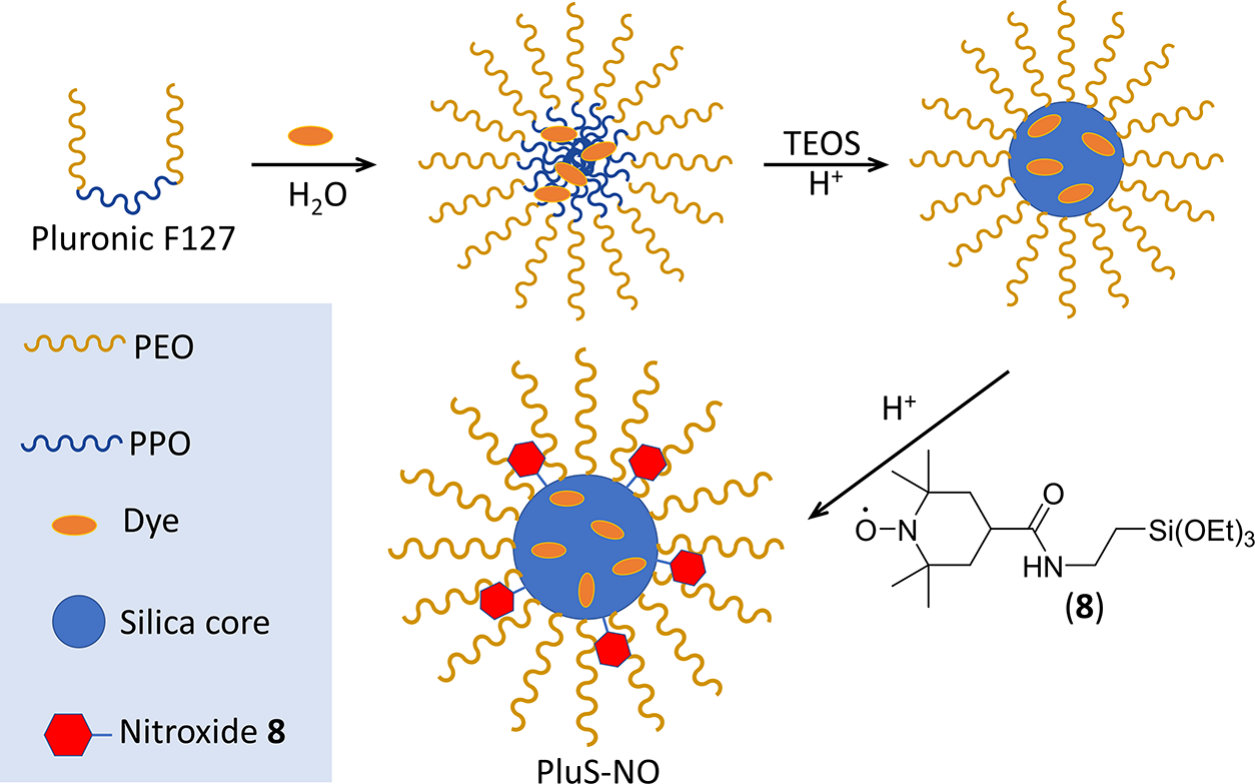
Investigated nitroxides and schematic structure of luminescent PluS–NO nanoparticles synthesized herein with the TEMPO derivative **8** and DEAC dyes embedded in the core (PEO = poly(ethylene oxide), PPO = polypropylene oxide).

The rational development of nitroxide-based nanoantioxidants requires the knowledge of the ability of the parent nitroxides to slow down the peroxidation of oxidizable substrates—reacting with ROO^•^ radicals, in fact, does not always guarantee antioxidant activity^32^—and how this reactivity is modified by the linkage to the nanomaterial. Unfortunately, little is known about this reaction in water, apart from the archetype nitroxide TEMPO.^19^ With this work, we aim at filling this knowledge gap for variously substituted nitroxides and for a novel nanoantioxidant, based on a silica core protected by PEG chains (Pluronic-silica nanoparticles, PluS) having nitroxide units covalently bound to the silica surface (PluS–NO) (Figure 2).^33,34^ PluS nanoparticles possess a small and monodisperse silica core (diameter 10 nm) and a hydrodynamic diameter of about 25 nm due to the intrinsic PEG shell, which results from the templating action of Pluronic F127 micelles during the one-pot synthesis.^33^ Silane derivatives can be co-reacted with the main silica precursor (tetraethoxysilane, TEOS) to yield luminescent nanolabels^35^ with phototherapeutic,^36^ sensing,^37^ cell penetration,^37^ and drug delivery^38^ abilities. A silane-functionalized nitroxide can be precisely localized on the surface of the silica core, allowing tuning of its activity.^39−41^ The results obtained with PluS–NO can be compared to those obtained with TEMPO (**1**) and nitroxides **2**–**7**, whose chain-breaking antioxidant activity in water solution was measured herein for the first time,^42^ allowing for a prompt and quantitative characterization of free and bound nitroxides.

## 2 Experimental Section

### 2.1 Materials and Methods

Analytical-grade solvents and commercially available reagents were used as received unless otherwise stated. Tetrahydrofuran (THF) was purified by distillation, 4,4′-azobis(4-cyanovaleric acid) (ABCV) was recrystallized from methanol, and Millipore grade water was used. Chromatographic purifications were performed using 70–230 mesh silica. ^1^H and ^13^C nuclear magnetic resonance (NMR) spectra were recorded on a Varian Mercury (400 MHz for ^1^H) spectrometer. Chemical shifts (δ) are reported in ppm relative to residual solvent signals in ^1^H and ^13^C NMR (^1^H NMR: 7.26 ppm in CDCl_3_; ^13^C NMR: 77.0 ppm in CDCl_3_,). ^13^C NMR spectra were acquired in the ^1^H broadband decoupled mode. Coupling constants are given in Hertz. Electrospray ionization mass spectrometry (ESI-MS) analyses were performed by direct injection of acetonitrile solutions of the compounds using a Waters ZQ 4000 mass spectrometer. Elemental analyses were performed on a Thermo Quest Flash 1112 series EA instrument. The exact mass was determined with a Waters Xevo G2-XS QTof with an ESI-APCI source.

### 2.2 Synthetic Procedures

#### 2.2.1 *N*-Butyl-2,2,6,6-tetramethyl-1-piperidinyloxy-4-carboxamide (**6**)

A solution of 100 mg (0.5 mmol) of 4-carboxy-TEMPO (**4**), 67.5 mg (0.5 mmol) of *N*-hydroxybenzotriazole, and 96 mg (0.5 mmol) of 1-(3-(dimethylamino)propyl)-3-ethylcarbodiimide hydrochloride (EDC·HCl) in 15 mL of CH_2_Cl_2_ was stirred at room temperature for 4 h. A solution of 1-butanamine (150 μL, 1.5 mmol) in 1 mL of CH_2_Cl_2_ was added, and the mixture was stirred at room temperature for 2 days. The solvent was removed in vacuo and the residue was purified by silica flash column chromatography (eluant, dichloromethane/methanol, 97:3), affording 106 mg (0.42 mmol) of **6** (yield = 86%). To render the paramagnetic compound suitable for NMR analysis it was quantitatively converted into the corresponding hydroxylamine derivative by adding a stoichiometric amount of phenylhydrazine in the NMR sample containing the nitroxide.^43^^1^H NMR (CDCl_3_, 400 MHz): δ 5.54 (bs, NH), 3.19–3.26 (m, 2H), 2.37–2.50 (m, 1H), 2.37–2.50 (m, 4H), 1.41–1.51 (m, 2H), 1.28–1.38 (m, 2H), 1.20 (s, 6H), 1.14 (s, 6H), 0.93 (t, *J* = 7.3 Hz, 3H). ^13^C NMR (CDCl_3_, 400 MHz): δ 174.6 (CO), 59.0 (C), 42.1 (CH_2_), 39.1 (CH_2_), 36.5 (CH), 31.9 (CH_3_), 31.6 (CH_2_), 20.0 (CH_2_), 19.5 (CH_3_), 13.7 (CH_3_). Electrospray ionization mass spectrometry (ESI-MS) *m*/*z*: 255 (M)^+^; 278 (M + Na)^+^; 294 (M + K)^+^. Exact mass (ESI-MS): 278.19656 (M + Na)^+^, expected: 278.19647 (C_14_H_27_N_2_O_2_Na).

#### 2.2.2 Silane Nitroxide (**8**)

**8** was synthesized using the procedure already reported in the literature (see the Supporting Information).^31^

#### 2.2.3 PluS–NO

We synthesized core–shell silica-PEG NPs by adapting previously reported procedures.^44^ Pluronic F127 (100 mg) and NaCl (68 mg) were carefully solubilized at 30 °C under magnetic stirring in 1.6 mL of 1 M acetic acid in a 20 mL glass scintillation vial. Upon complete solubilization (ca. 1 h), the desired amount of silanized dye (DEAC-silane, 6.5 mmol, 0.8% vs mol TEOS, prepared following an already published procedure)^33^ was added to the micellar suspension. TEOS (180 μL, 0.81 mmol) was then added to the resulting aqueous homogeneous solution and left to react overnight. After 24 h the antioxidant (TEMPO silane derivative **8**, 8 mmol, 1% vs mol TEOS) was added to the dispersion, followed by addition of trimethylsilyl chloride (TMSCl, 10 μL, 0.08 mmol) after 6 h. The mixture was kept under stirring for 20 h at 30 °C before dialysis workup. The dialysis purification steps were carried out vs water on a precise amount of NP solution (800 μL), finally diluted to a total volume of 5 mL with water. The dimensions, measured by transmission electron microscopy (TEM) and dynamic light scattering (DLS), respectively, are 10 nm diameter of the core and 29 nm hydrodynamic diameter (intensity mean *D*_H_ obtained by DLS) and did not change over 2 years storage at room temperature in the dark. Absorption and emission properties (see the Supporting Information) were similar to those previously reported for analogous nanoparticles without nitroxide.^33^

### 2.3 Electron Paramagnetic Resonance (EPR) Spectroscopy Studies

The EPR spectra were collected at 25 °C with a MiniScope MS 5000 (Magnettech) in glass capillary tubes. The concentration of nitroxide bound to the nanoparticles was determined by comparing the double integral of its EPR spectrum to that of reference nitroxide **2**. Spectral simulation was performed using EasySpin software with the graphical interface SimLabel.^45,46^

### 2.4 Autoxidation Studies

Oxygen consumption during autoxidation experiments was measured with an optical oxygen meter (Firesting O_2_, Pyro Science GmbH).^47^ Typical samples consisted of ABCV 27 mM, NaOH 54 mM, THF (10 or 25% v/v), pH 7.4 0.1 M phosphate buffer, 30 °C. Using the α-tocopherol hydrosoluble analogue Trolox as a reference antioxidant (having *n* = 2), the rate of radical initiation was calculated as *R*_i_ = 1.6 × 10^–9^ M s^–1^ for [ABCV] = 27 mM by the relation *R*_i_ = 2[Trolox]/τ, where τ is the duration of the inhibition period. This equation also provided the stoichiometry of the antioxidant *n* (see Table 1).^48^ Numerical analysis of O_2_ consumption traces was performed following a previously reported procedure^18,19^ using Copasi software,^49^ freely available on the Internet, using the *k*_p_ and *k*_t_ values (30 °C) of the THF autoxidation reported in the literature.^19,50,51^ The complete procedure, examples of the experimental and simulated O_2_ traces (Figure S9), and a detailed list of obtained rate constants (Table S2) are reported in the Supporting Information.

**Table 1:** Antioxidant Activity of the Investigated Nitroxides Studied by the Inhibited Autoxidation Method^a^

*k*_inh_ (M^–1^ s^–1^)			*n*	*E*° (V)
**1**	(5.1 ± 1.5) × 10^6^	(4.6 × 10^6^;^b^ 2.8 × 10^7^^c^)	3.8 ± 0.4	0.722^d^
**2**	(1.1 ± 0.5) × 10^6^	(3.3 × 10^6^)^c^	4.7 ± 0.7	0.810^d^
**3**	(5.4 ± 1.5) × 10^5^	(1.0 × 10^6^)^c^	4.5 ± 0.6	0.826^d^
**4**	(3.7 ± 1.0) × 10^5^		1.9 ± 0.3	0.82^e^
**5**	(1.1 ± 0.4) × 10^5^		1.8 ± 0.1	0.88^e^
**6**	(1.9 ± 0.5) × 10^5^		1.9 ± 0.3	
**7**	(5.6 ± 1.2) × 10^4^	(2.8 × 10^5^)^c^	1.5 ± 0.2	0.913^c^
PluS–NO	(1.5 ± 0.4) × 10^5^		3.7 ± 0.5	
			111 ± 15^f^	
Trolox	(2.6 ± 0.7) × 10^5^		2^g^	

a Rate constant of the reaction with peroxyl radicals (*k*_inh_) with literature values in parenthesis, stoichiometric coefficient (*n*) and redox potential for the oxoammonium/nitroxide couple (H_2_O vs normal hydrogen electrode (NHE)).

b From ref (19).

c Reaction with *^t^*BuOO^•^ obtained by pulse radiolysis, from ref (15).

d From ref (15).

e From ref (53).

f Overall *n* of the PluS–NO nanoantioxidant.

g Reference value, from ref (32).

## 3 Results and Discussion

### 3.1 Synthesis of PluS–NO Nanoparticles and EPR Characterization

**Figure 3:**
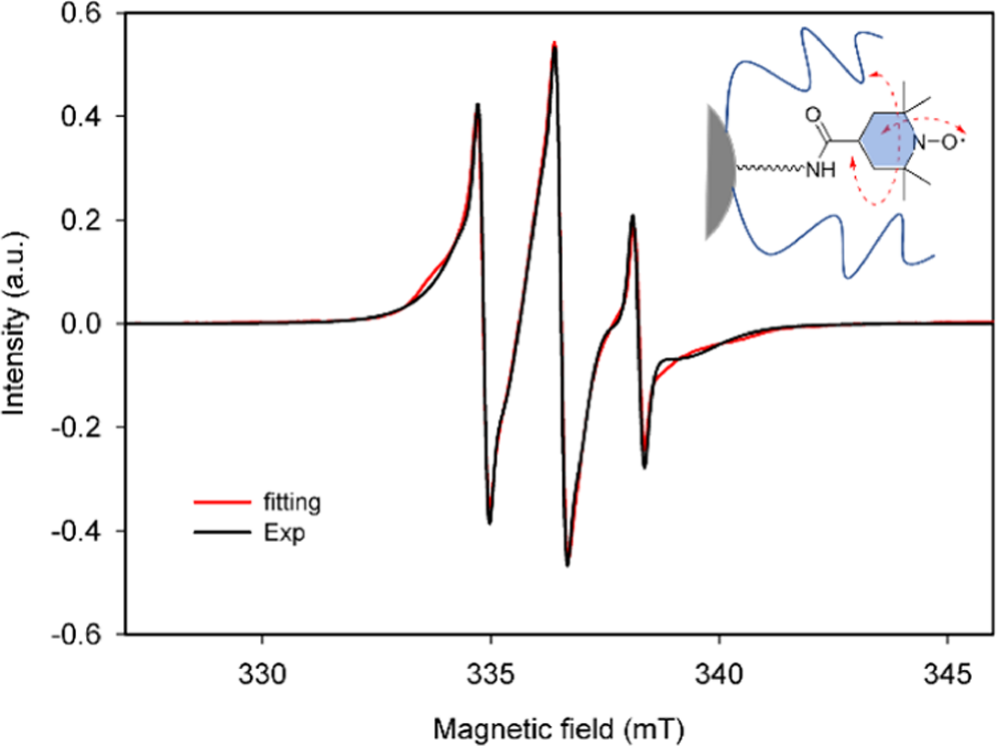
Experimental and simulated EPR spectrum of PluS–NO (2 × 10^–5^ M in water).

Core–shell silica-PEG nanoparticles were prepared by hydrolysis/condensation of tetraethoxysilane (TEOS) under acidic conditions in a micellar solution of Pluronic F127, a triblock polyethylene oxide, i.e., the poly(ethylene glycol))–polypropylene oxide copolymer as already reported (see Figure 2).^33^ The desired amount of the silanized dye (7-(diethylamino)-*N*-(3-(triethoxysilyl)propyl)coumarin-3-carboxamide that we indicate here as DEAC and antioxidant **8** were added to the micellar suspension before and after the condensation step, respectively. After the dialysis workup, we obtained nanoparticles having the expected hydrodynamic diameter DH of ca. 29 nm, with a concentration of 2 × 10^–5^ M. The electron paramagnetic resonance (EPR) spectrum of PluS–NO reported in Figure 3 was the typical spectrum of nitroxides in the slow-motion regime, characterized by broadened lines and uneven heights indicative of an increased correlation time τ_c_ (i.e., the time required to rotate one radian, ∼57°) of the radical.^52^

Numerical fitting of the experimental spectra assuming isotropic motion provided an estimate of 4 × 10^–9^ s for τ_c_, which is a much larger value with respect to τ_c_ in solution (≈10^–11^ s) (see the Supporting Information for the comparison with the EPR spectrum of **6**).^52^ The restricted mobility demonstrates that the radical is anchored to a rigid matrix, surrounded by a soft, longer shell (see Figure 3), and is similar to the results obtained in the case of nitroxides linked to the surface of gold nanoparticles.^52^

The area of the EPR spectrum provided the concentration of the nitroxide in the sample as (6.0 ± 0.2) × 10^–4^ M, which divided by the nanoparticle concentration afforded an average coverage of 30 nitroxides per nanoparticle. PluS–NO are both colloidally and chemically very stable in water, as proven by the constant *D*_H_ and EPR signal of the nitroxide over at least 1 year at 5 °C.

### 3.2 Inhibited Autoxidation Studies

The antioxidant activity of nitroxides **1**–**7** and PluS–NO were investigated by studying their effect on tetrahydrofuran (THF) autoxidation initiated by the azo-initiator 4,4′-azobis(4-cyanovaleric acid) (ABCV) at 30 °C in phosphate buffer at pH 7.4. The rate of THF autoxidation was determined by measuring the O_2_ consumption in a close reaction vessel, as shown in Figure 3.

**Scheme 1:**
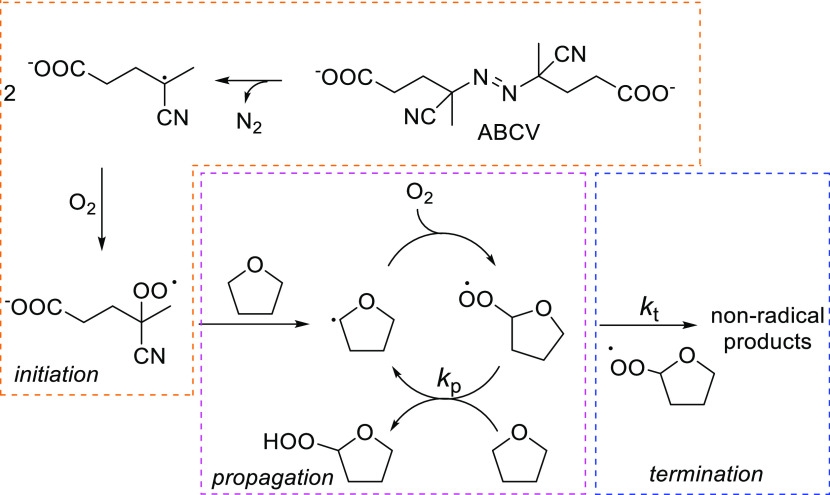
Mechanism of THF Autoxidation

THF autoxidation follows the typical mechanism of biologically relevant organic compounds, consisting of the initiation, propagation, and termination steps (Scheme 1), which involve carbon (R^•^) and oxygen-centered peroxyl (ROO^•^) radicals.^50^

**Figure 4:**
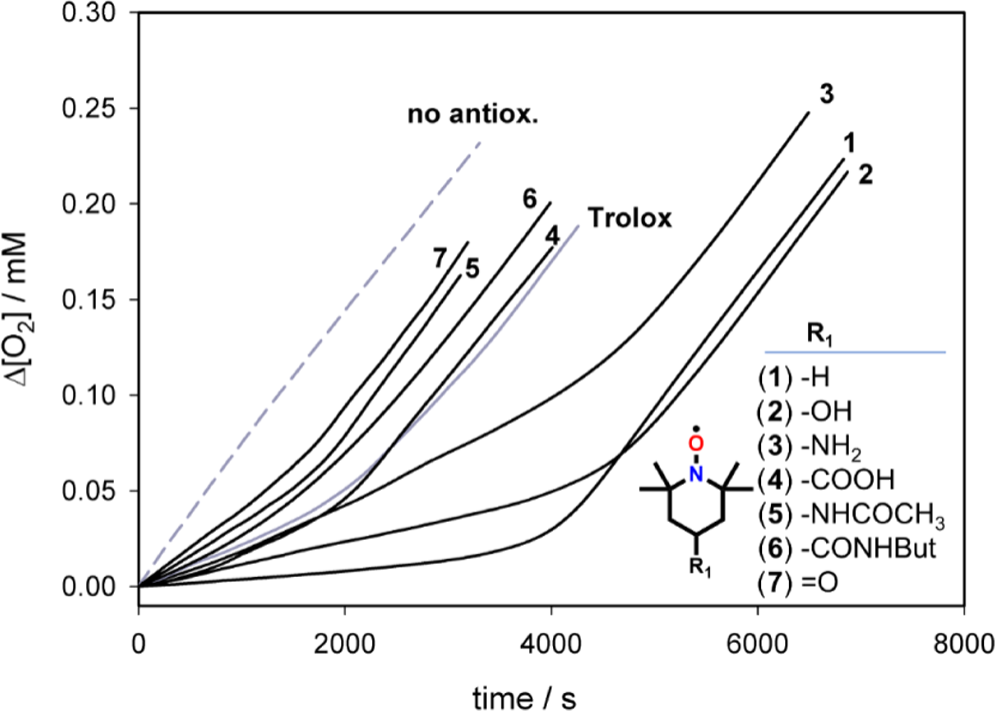
Oxygen consumption measured during the autoxidation of THF (3.1 M) initiated by ABCV (27 mM) in phosphate buffer (pH 7.4) at 30 °C inhibited by Trolox and the title nitroxides (all 1.4 μM).

In the absence of antioxidants, O_2_ consumption is fast and linear (see the dashed gray line in Figure 4), while upon the addition of an antioxidant, the O_2_ consumption is reduced. The slope of the inhibition period is inversely proportional to the rate constant of the reaction with radicals, while the duration of the inhibition depends on the number of radical trapped by each antioxidant (stoichiometric coefficient, *n*).^48^

**Scheme 2:**
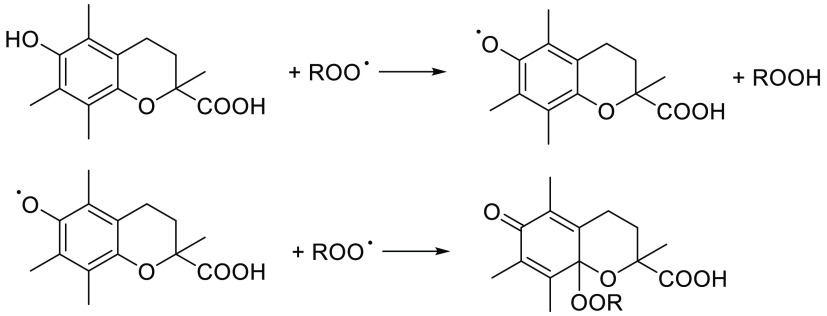
Antioxidation Mechanism of Trolox

The mechanism of the antioxidant effect of the nitroxides is different from that of phenolic antioxidants. Indeed, the latter ones typically have *n* = 2 deriving from the consecutive reaction with two peroxyl radicals, as exemplified in Scheme 2 for Trolox,^32^ which is used in our study as the reference antioxidant. Instead, stoichiometries of nitroxides have been found between 1 to values as big as 6 because of their possibility to participate in the cyclic regeneration mechanism shown by reactions 1–4.^19^ First, ROO^•^ radicals react with nitroxides by a proton-coupled electron transfer (reaction 1), forming an oxoammonium cation and a hydroperoxide. Then, the oxoammonium cation reacts with the available reductants present in the system (such as THF)^19^ forming hydroxylamine (reaction 2), which regenerates the nitroxide by the reaction with another ROO^•^ radical (reaction 3). The duration of the inhibition depends on the competition between the reaction with ROO^•^ and the one with R^•^ (reaction 4), a termination step yielding an inactive alkoxyamine.^19,53^
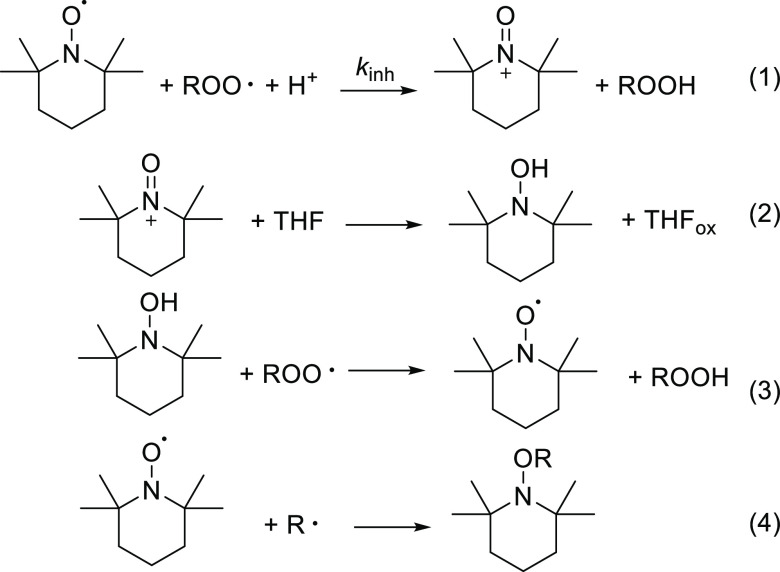


Therefore, *n* = 1 when reaction 4 is much faster than reaction 1, while *n* > 1 indicates an increasing weight in reaction 1 and of the regeneration cycle. The values of *k*_inh_ were determined by numerical analysis of the O_2_ consumption plots using the rate constants for reactions 2–4 reported in the literature as constraints, and are reported in Table 1.^19^ The highest activity is exhibited by unsubstituted TEMPO (**1**), while heteroatoms in position 4 reduce *k*_inh_ by decreasing the electron donation of the R_2_NO^•^ moiety via inductive effects. The *k*_inh_ values for **1**, **2**, and **3** are in reasonable agreement with the rate constants of the reaction with *^t^*BuOO^•^ radicals measured by Goldstein and Samuni by pulse radiolysis at pH 7,^15^ and with the *k*_inh_ of **1** reported by Pratt and co-workers, measured by studying THF autoxidation (see Table 1).^19^ This proves the appropriateness of the oximetry technique employed, which hence shows reasonable accuracy in the results, with the important advantage of being easily extensible to materials not suitable for conventional spectroscopic assays (such as nanoantioxidants, see below).

To investigate the mechanism underlying reaction 1 in deeper detail, Marcus theory of outer-sphere electron transfer (ET) processes was employed.^54^ The rate constant *k*_inh_ for the reaction between a nitroxide and the peroxyl radical of THF can be written as in Equation 1, where *Z* is the preexponential factor, λ is the reorganization energy of both the substrate and solvent, and Δ*G*°′ is the corrected free energy change of the reaction.
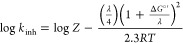
1Δ*G*°′ is defined as Δ*G*°′ = Δ*G*° + *A*, where Δ*G*° is the standard free energy change for the ET reaction, Δ*G*° = −23.06 [*E*°(ROO^•/–^) – *E*°(NO^+/•^)], and the term *A* represents the correction for the electrostatic free energy change (*A* ≈ −0.5 kcal/mol).^55^

**Figure 5:**
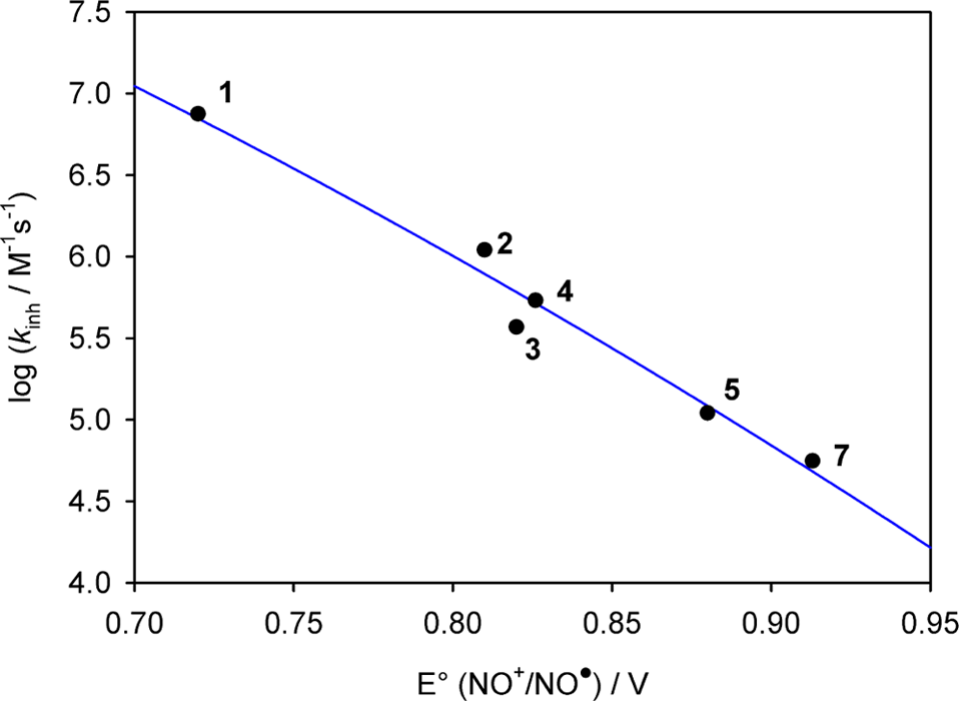
Marcus relationship between log *k*_inh_ and *E*°(NO^+/•^), the line represents the fitting to eq 1.

Upon setting *Z* to the typical value for an adiabatic ET (*Z* = 10^11^ M^–1^ s^–1^),^55^ the fit of eq 1 to experimental log (*k*_inh_) vs *E*°(NO^+/•^) data points, reported in Figure 5, affords *E*°(ROO^•/–^) = 0.62 V (vs NHE) and λ = 19 kcal/mol. These results are in reasonable agreement with the reported data of the redox potential of alkylperoxyl radicals (0.71 V vs NHE for *^t^*BuOO^•^)^56^ and with the reorganization energy for ET from ferrocenes to CH_3_OO^•^ (λ = 33 kcal/mol),^57^ suggesting that the mechanism is a stepwise electron transfer–proton transfer (ET–PT) where ET is the rate-limiting step.

Overall, these experiments show that except for carboxy-TEMPO **7**, all piperidine-derived nitroxides represent a good antioxidant tag for further functionalization strategies as they have *k*_inh_ values larger or comparable to that of the reference antioxidant Trolox. Nevertheless, as the best ROO^•^ trapping is shown by **1**, it may be suggested that the structure of nitroxide antioxidants can be optimized for instance by increasing the distance with the functional group in the 4 position by alkyl substituents.

Proper functionalization of nitroxides allows their covalent binding to nanostructures to yield multifunctional nanoantioxidants. The study and the comparison of free and bound active species can evidence the possible variations in their reactivity when included in nanostructures.

**Figure 6:**
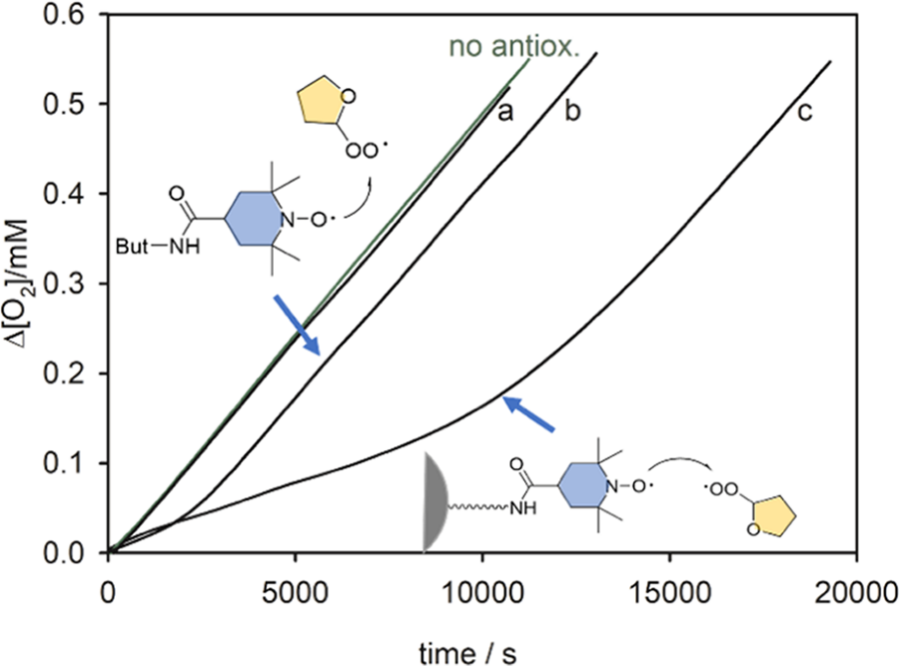
Oxygen consumption during the autoxidation of THF (1.6 M) initiated by ABCV (50 mM) at 30 °C in the presence of: (a) bare nanoparticles (0.25 μM); (b) nitroxide **6** (5 μM); and (c) PluS–NO (0.50 μM corresponding to [nitroxide] = 15 μM).

In this framework, we have investigated the antioxidant activity of PluS nanoparticles bearing the silanized TEMPO derivative **8** covalently bound on the surface of the silica core. The data obtained are shown in Figure 6 together with compound **6** that is structurally the most similar to the non-silanized free antioxidant counterpart. PluS–NO inhibited THF autoxidation (see trace c), whereas unfunctionalized ones had no effect (trace a). The measured rate constant of the reaction with ROO^•^ radicals was (1.5 ± 0.4) × 10^5^ M^–1^ s^–1^ while the number of radical trapped was 3.7 ± 0.5 per nitroxide unit. From the perspective of molecular nitroxide, these results show that—even upon binding to the silica surface of PluS nanoparticles—its antioxidant activity is largely preserved, with a *k*_inh_ value similar to that of model nitroxide **6** and only slightly lower than that of Trolox. In addition, a 2-fold higher stoichiometric coefficient *n* is observed when bound to the silica surface, possibly suggesting a smaller tendency of alkyl radicals to add to the bound nitroxide (i.e., by reaction 4), representing a specific advantage of PluS–NO over the molecular counterpart. From the perspective of the nanoantioxidant considered as a whole, PluS–NO displays ≈60-fold increased inhibition of THF autooxidation compared to molecular nitroxide **6**, owing to the local accumulation of active species (about 30 nitroxides per particle) and to the enhanced number of trapped radicals by each nitroxide. In addition, we inserted ≈36 DEAC dyes per NP covalently linked into the core, adding the functionality of fluorescence labeling to the antioxidant activity. DEAC photophysics does not suffer from the presence and reactivity of nearby nitroxides, featuring similar absorption and emission (λ_max_ = 415 and 472 nm, respectively, Figure S2) properties as those of previously reported DEAC-doped PluS NPs without nitroxides.^33^ Finally, PluS NPs have previously been reported to be suitable for active or passive targeting of various bio-targets, including specific transport proteins, cancer biomarkers,^58^ and sentinel lymphnodes,^59^ revealing the potential of nanoantioxidants based on PluS–NO as specific multifunctional agents.

## 4 Conclusions

Rational design of nitroxide-based nanoantioxidants to be used in complex water-based environments requires—among other things—a method to quantitatively compare their ability to slow down the peroxidation of oxidizable substrates with the parent molecular nitroxides. To this goal, we have investigated the antioxidant activity in water of nitroxides **2**–**7** for the first time and compared them with the well-known TEMPO and the reference antioxidant Trolox. The results reveal that all nitroxides are good antioxidants with inhibition constants values similar to or larger than that of Trolox, except for **7**, while the best ROO^•^ trapping ability is shown by **1**. The method is successfully applied to nanoantioxidants PluS–NO, obtained by locating silanized nitroxides on the silica surface of PluS NPs. These NPs preserve nitroxide reactivity, showing nearly identical *k*_inh_ values with respect to the unbound nitroxide **6**; in addition, the number of radicals trapped by every single silica-bound nitroxides is doubled, while the whole PluS–NO nanoantioxidant shows a trapping ability toward the radicals that is ≈60-fold higher compared to the parent nitroxide **6**.

This study is important to understand how to optimize the structure of nitroxide antioxidants to allow their chemical binding in a nanostructure without affecting their properties. As the antioxidant activity of TEMPO derivatives is decreased by any functional group in the 4 position, these results call for the synthesis of novel nitroxides having an optimized structure, for instance, with the substituent separated by an alkyl chain. Moreover, the importance of the regeneration cycle in determining the duration of the inhibition suggests that nitroxides should be used in the presence of sacrificial reductants to fully exhibit their activity.

PluS–NO also enjoys the versatility of the PluS–NP architecture, in particular tunable fluorescence (including cascade FRET for high brightness and Stokes-shift for NIR emission) and bio-targeting capability. These results show that with proper knowledge of the antioxidant activity and a rational design, silica-bound TEMPO radicals can be suitable building blocks for the development of new multifunctional nanoantioxidants, which could find application as redox modulators even in biological systems.

## Supporting Information Available

Details of the synthesis of **6** and **8**, TEM images, absorption and emission spectra of PluS–NO, numerical fitting of EPR spectra using Simlabel software, and numerical fitting of O_2_ consumption plots (PDF)
