# Supplementary material for: Nitroxides as Building Blocks for Nanoantioxidants
Source: ACS Appl Mater Interfaces. 2021 Jun 22;13(27):31996–2004. doi: 10.1021/acsami.1c06674 (PMC8289242; doi:10.1021/acsami.1c06674)
Supplement: Supplementary file 1 — am1c06674_si_001.pdf [file am1c06674_si_001.pdf]

Supporting Information

Nitroxides as building blocks for nanoantioxidants

Damiano Genovese,[a] Andrea Baschieri,[b] Danilo Vona,[c] Ruxandra Elena Baboi,[a] Fabio Mollica,[a] Luca Prodi,[a]

Riccardo Amorati*[a] and Nelsi Zaccheroni,[a]

[a] Department of Chemistry "G. Ciamician", University of Bologna, via Selmi 3 and via San Giacomo 11, 40123, Bologna, Italy.

[b] Istituto per la Sintesi Organica e la Fotoreattività, Consiglio Nazionale delle Ricerche (ISOF - CNR), via Gobetti 101, 40129 Bologna, Italy.

[c] Department of Chemistry, University of Bari, via Orabona 4, I-70126 Bari, Italy

Content | Page

Content	Page
Synthesis and FT-IR spectrum of silane-nitroxide **8**	S2
Absorption and emission spectra of PLUS-NO	S3
TEM images (magnifications in the inset) of PluS-NO NPs	S3
NMR and ESI mass spectra of compound **6**	S4 -S6
Numerical fitting of experimental X-band EPR spectra by Simlabel software	S7-S8
Numerical simulation of autoxidation of THF inhibited by nitroxides	S9-S10
References	S10

The silane nitroxide (8) was synthesized using the procedure already published in literature (ref S1). In a three-neck round-bottom flask 4-carboxy-TEMPO (50 mg, 0.25 mmol) was added to a solution of APTES (58 µL, 0.25 mmol), EDC·HCl (48 mg, 0.25 mmol), and DMAP (1.2 mg, 0.025 mmol) in dry CH_2Cl_2 (10 mL) at 0°C and the resulting mixture was stirred at room temperature for 48 h. The reaction was monitored by TLC and GC–MS until the starting reagents had disappeared. The reaction mixture was quenched with water and extracted with CH_2Cl_2 (2x50 mL). The organic layers were combined, and the solvent was evaporated by a rotary evaporator. The pale orange oil residue was purified by column chromatography using ethyl acetate as the eluent to give an orange-colored oil (61% yield).

MS (EI, 70 eV) m/z (%): 403 [M+], 373 (23), 357 (39), 327 (21), 271 (21), 202 (100), 160 (45), 124 (80), 107 (32), 79 (13). FTIR (KBr): ν = 3320, 2970, 2920, 1650 (C=O), 1550 (NH), 1450 (CH_3), 1380 (NO·), 1325, 1102 (SiO), 1081 cm^{-1}. Elemental analysis calc.d (%) for $C_{19}H_{39}N_2O_5Si$ (403.6): C 56.54, H 9.74, N 6.94; found: C 56.79, H 9.85, N 7.05.

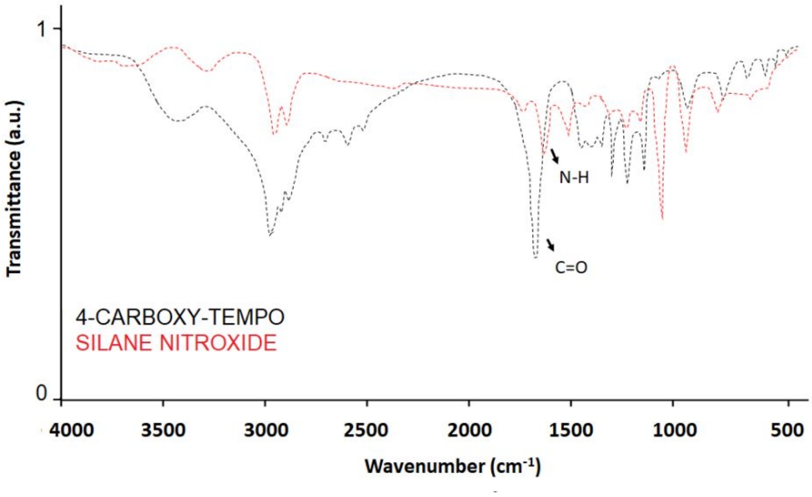

Figure S1. FT-IR spectrum of a KBr pellet of nitroxide **8** (silane nitroxide) and nitroxide **4** (4-carboxy-TEMPO).

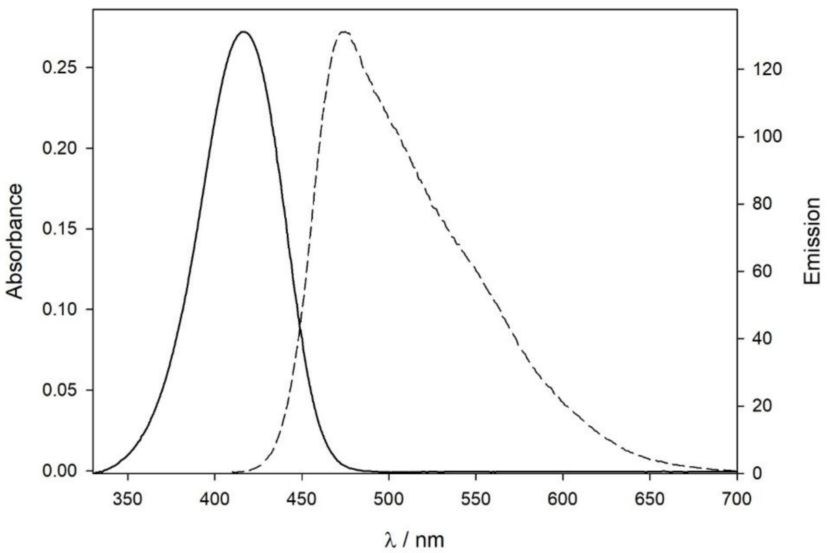

Figure S2. Absorption (continue line) and emission (dashed line, λ_{ex} = 400 nm) spectra of PluS-NO nanoparticles (2.0 x 10^{-7} M in H_2O). (Data obtained with a Perkin-Elmer Lambda 45 spectrophotometer and an Edinburgh FLS920 equipped with a photomultiplier Hamamatsu R928P)

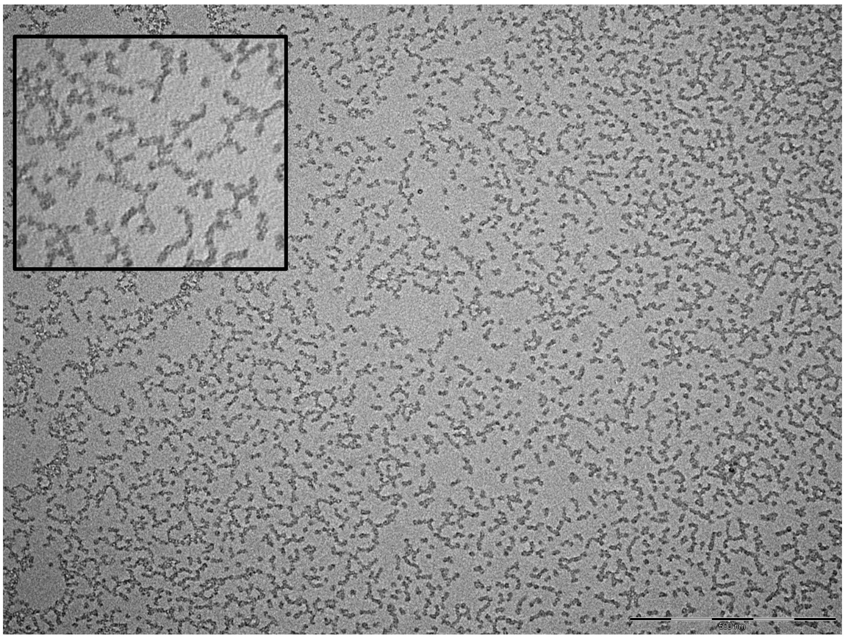

Figure S3. TEM images (magnifications in the inset) of PluS-NO NPs (scale bar 500 nm). Only the dense silica core of diameter = 10 nm ± 1 can be seen with this technique. (Images taken with a Philips CM 100 TEM operating at 80 kV)

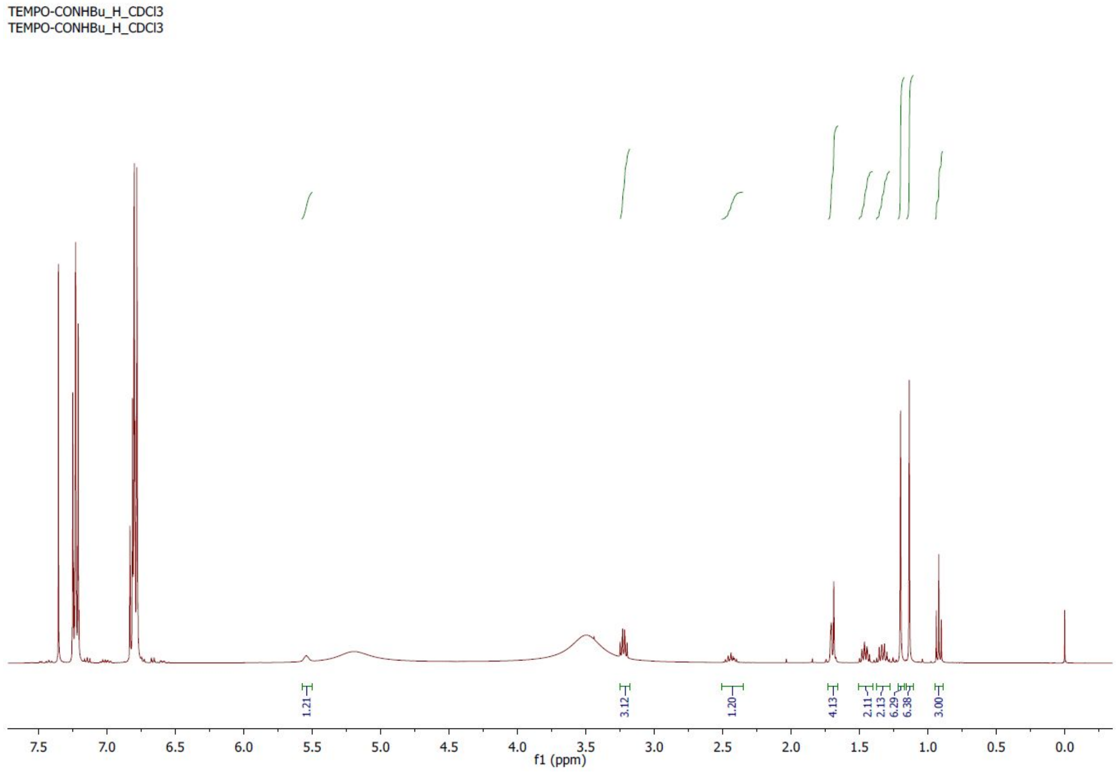

Figure S4. ^{1}H NMR spectrum of compound **6**

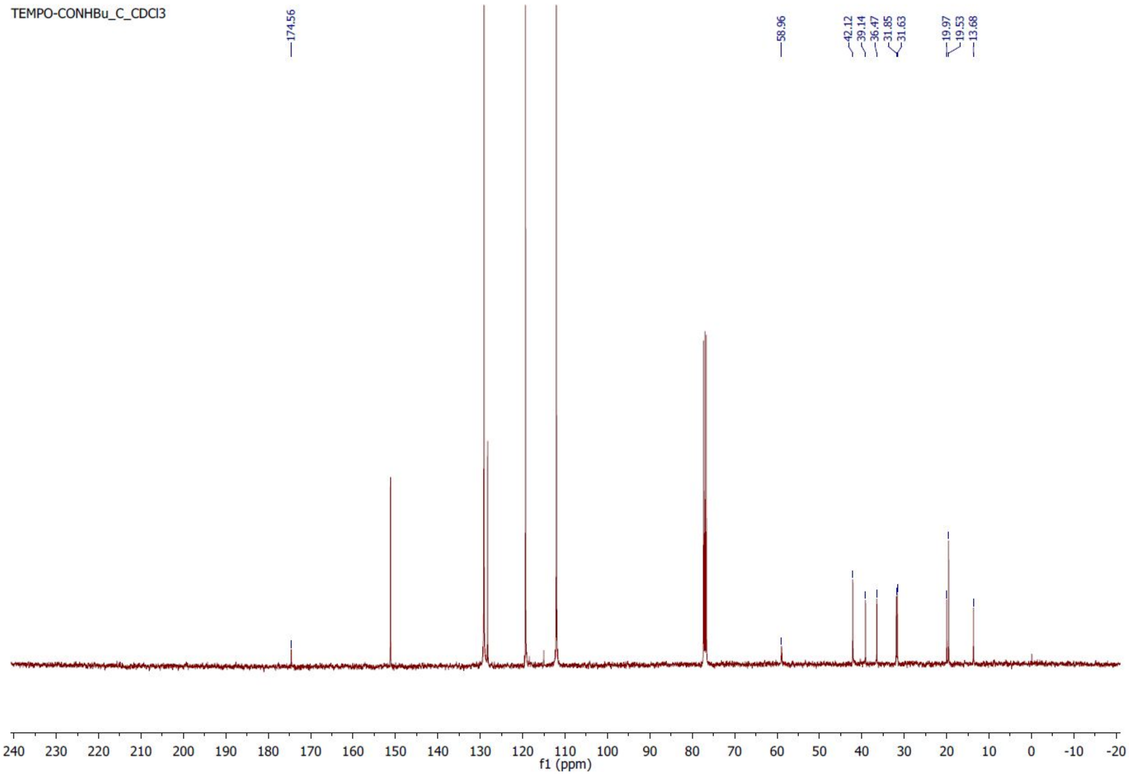

Figure S5. ^{13}C NMR spectrum of compound **6**

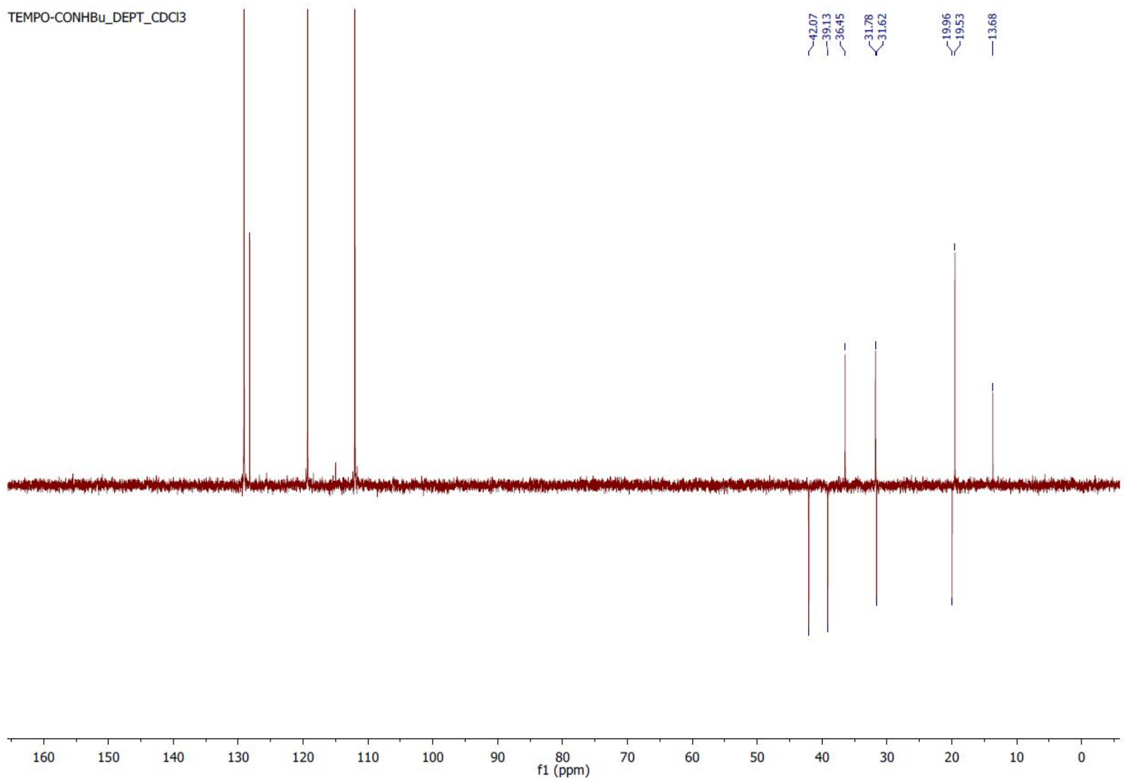

Figure S6. DEPT 135 spectrum of compound **6**

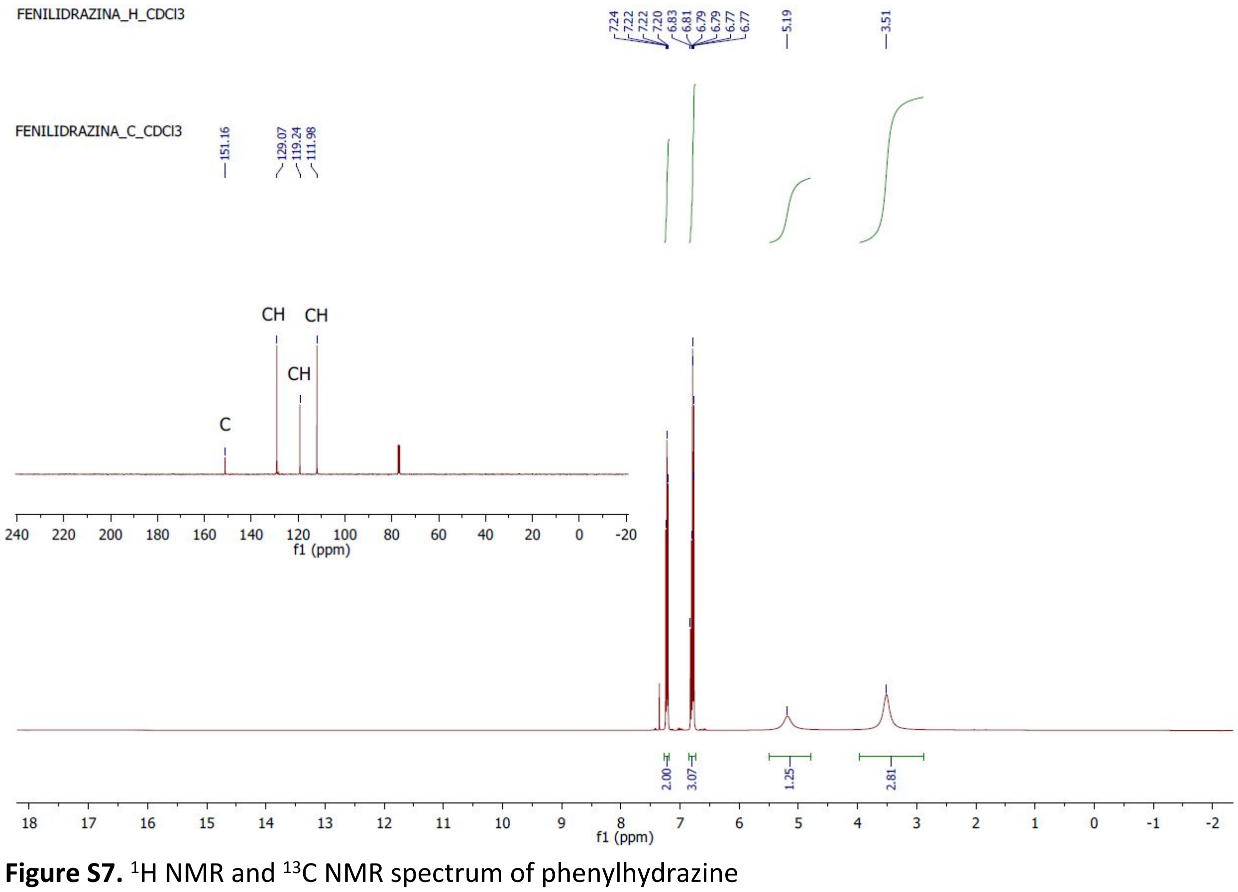

Figure S7. ^{1}H NMR and ^{13}C NMR spectrum of phenylhydrazine

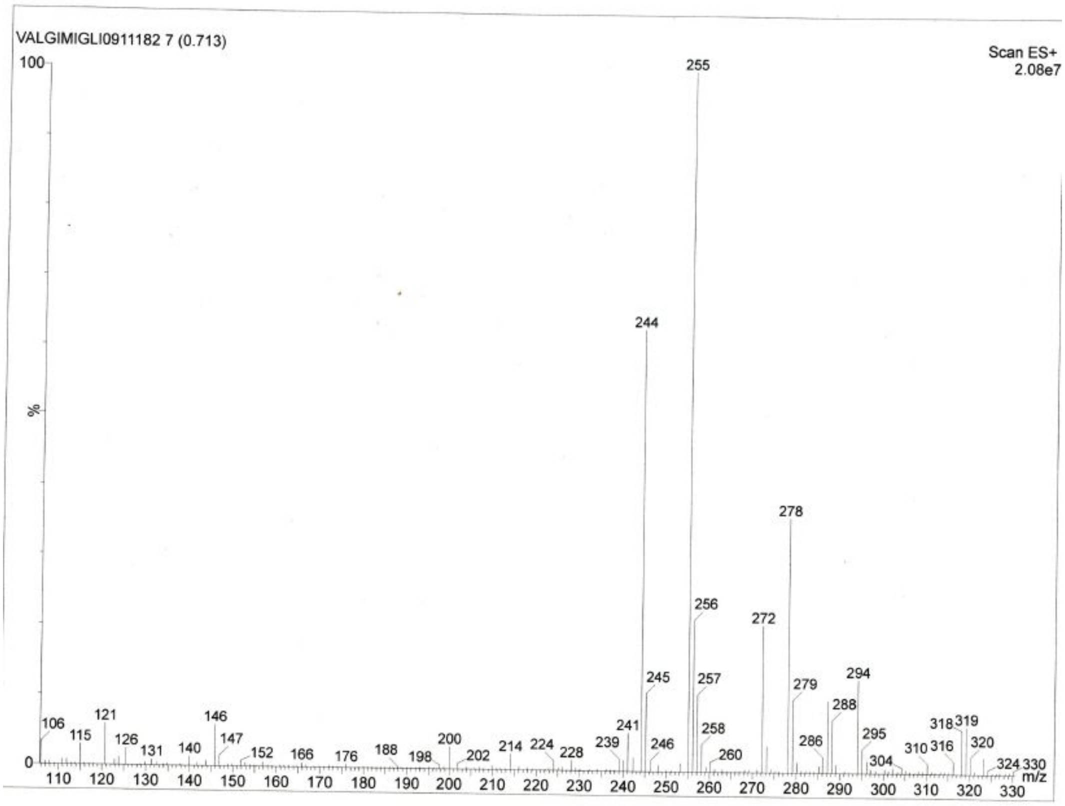

Figure S8. ESI^{+} mass spectrum of compound **6**

Numerical fitting of experimental X-band EPR spectra by Simlabel software

1) Results of the numerical simulation of the experimental spectrum of Plus-NO (black).
The simulated spectrum contains two components. The first one has a low correlation time and an abundance of 92.6%, and was identified as the bound nitroxide. The second one has a higher correlation time, an abundance of 7.4 %, and was interpreted as a trace of unreacted nitroxide adsorbed on the surface.

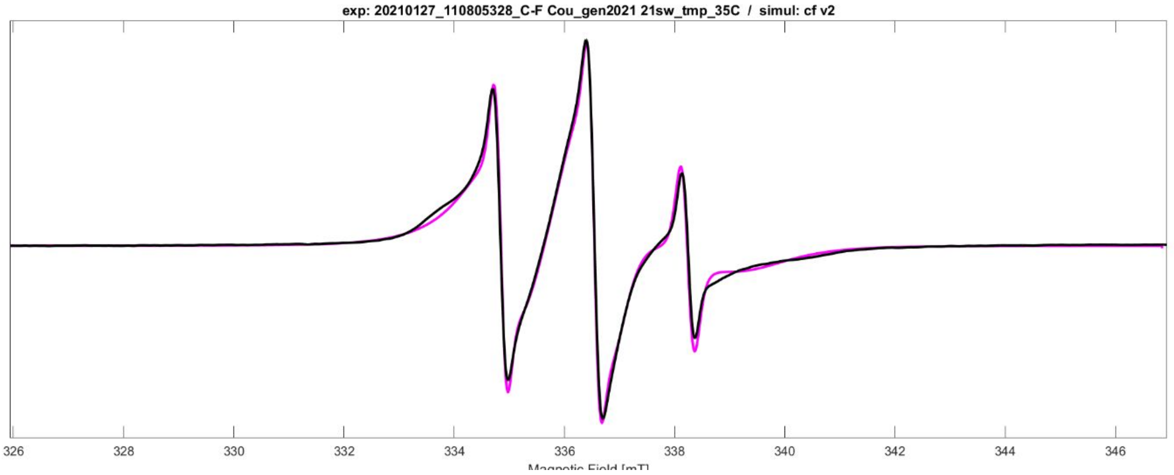

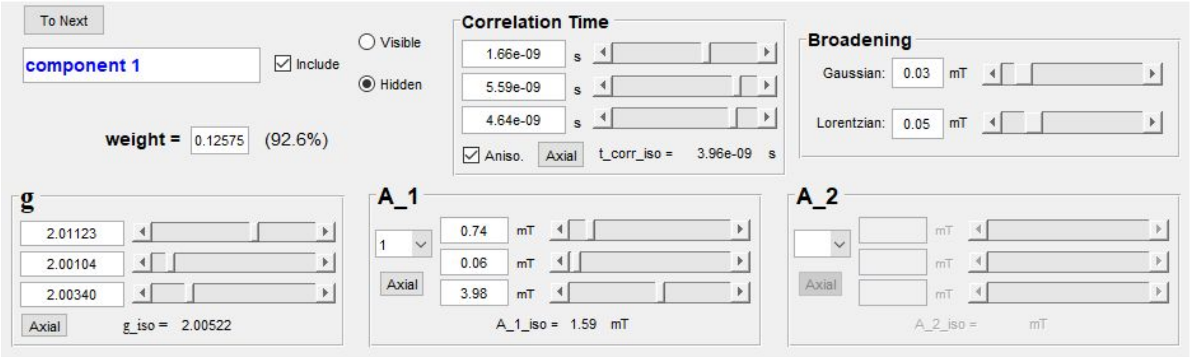

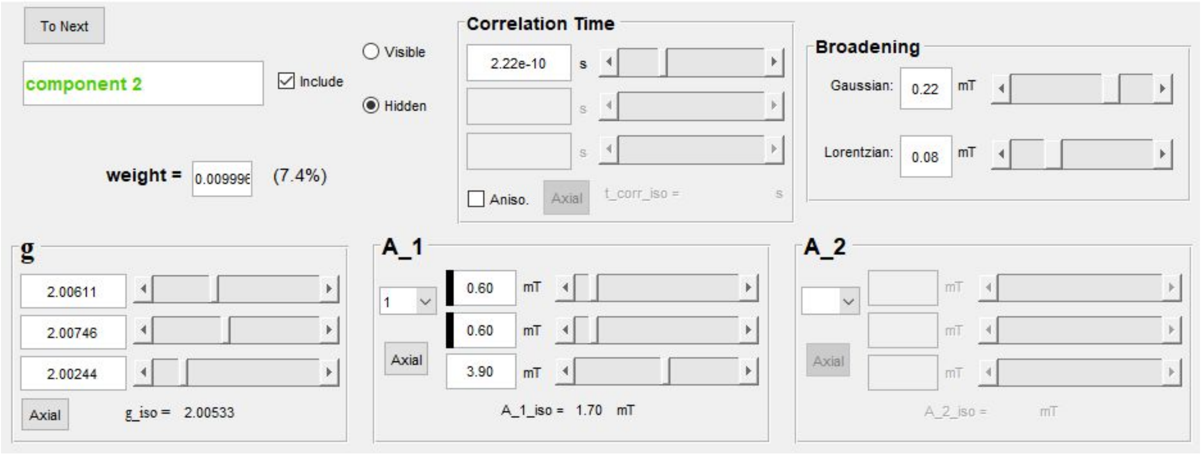

2) Results of the numerical simulation of the experimental spectrum of nitroxide **6**. The low correlation time is consistent with a dissolved nitroxide.

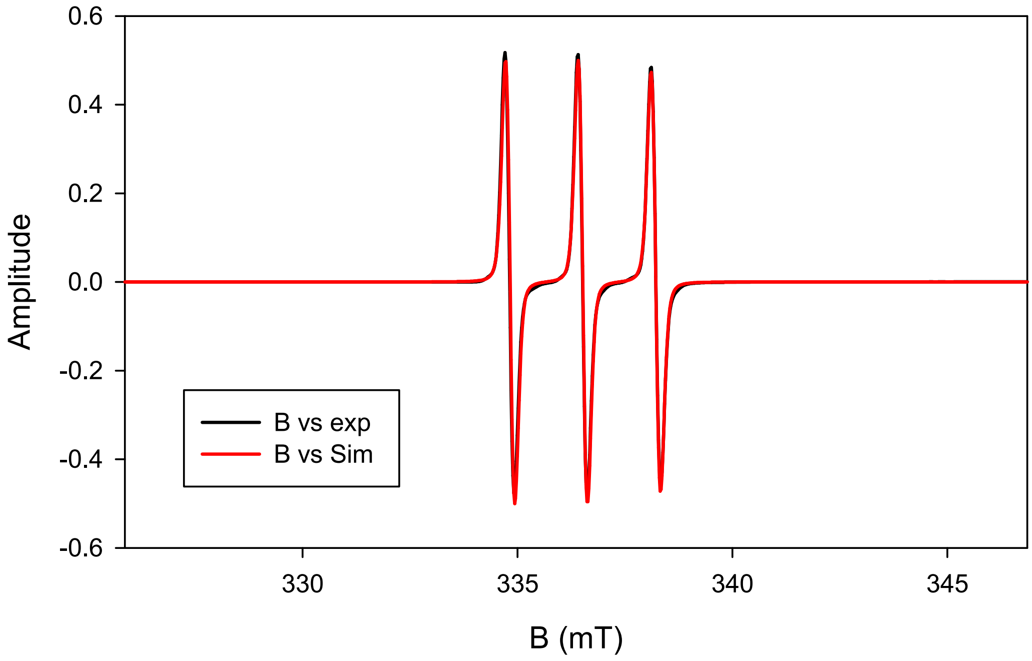

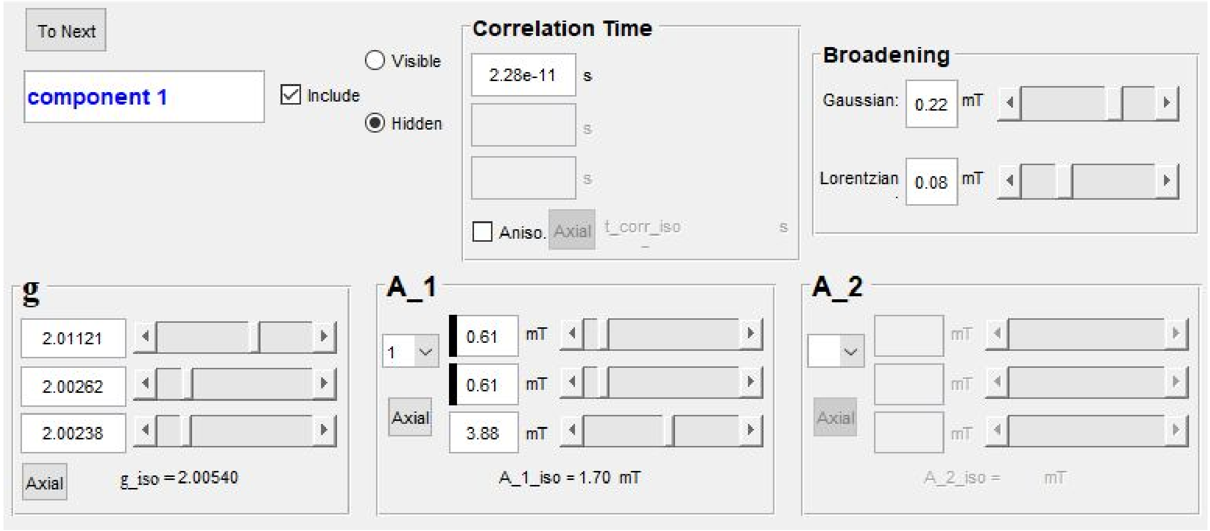

Numerical simulation of autoxidation of THF inhibited by nitroxides

Table S1. Reaction scheme and rate constants. R• = alkyl radicals, ROO• = peroxyl radicals, RH = THF, NO• = nitroxide; NOx = oxoammonium cation, NOH = hydroxylamine.

**Reaction**	**Rate constant**	**note**
Initiator → R•	Ri = 1.6e–9 Ms^{-1}	Experimentally determined
R• + O_2 → ROO•	*k* = 4e9 $M^{-1}s^{-1}$	Ref S2
ROO• + RH → ROO + R•	*k* = 4.8 $M^{-1}s^{-1}$	Ref S3
ROO• + ROO• → Inactive Products	*k* = 3.3e7 $M^{-1}s^{-1}$	Ref S3
NO• + R• → NOR	*fitted*	
NO• + ROO• → NOx + ROOH	*Fitted*	
NOx + RH → NOH + RHox	*Fitted*	
NOH + ROO• → NO• + ROOH	fitted	

Table S2. Results of numerical simulations.

**4X-TEMPO**	*k*(NO•+ROO•) $M^{-1}s^{-1}$	*k*(NO•+R•) $M^{-1}s^{-1}$	*k*(NOx+RH) $M^{-1}s^{-1}$	*k*(NOH+ROO•) $M^{-1}s^{-1}$
H (**1**)	5.1e6	2.0e11	2e-3	3.8e6
OH (**2**)	1.1e6	2.7e10	0.01	2.5e6
NH_2 (**3**)	5.4e5	1.5e10	0.02	1.0e6
COOH (**4**)	3.7e5	4.0e10	6e-4	4.3e6
$NHCOCH_3$ (**5**)	1.1e5	2.6e10	0.01	2.3e6
CONHBut (**6**)	1.9e5	2.7e10	5e-4	4.5e6
=O (**7**)	5.6e4	1.9e10	3.6e-3	2.0e6
PLUS-NO	1.5e5	1.8e10	2.4e-4	1.2e6

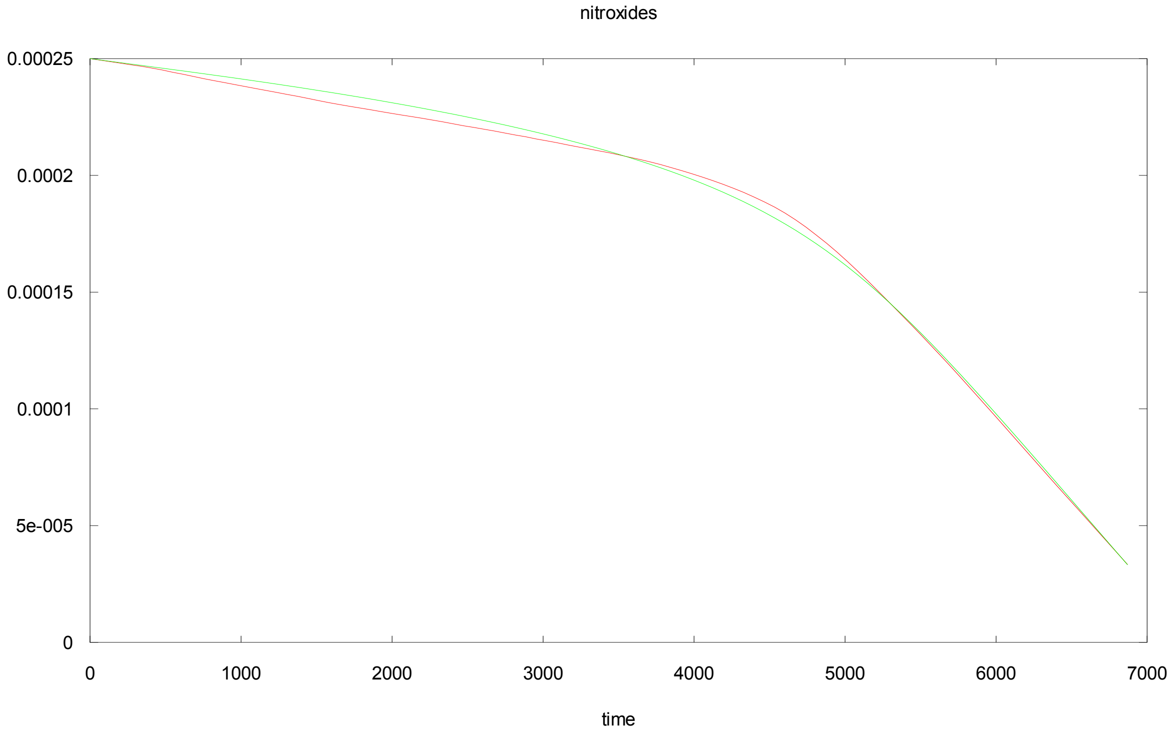

Figure S9. Example of result obtained by the numerical simulation. Autoxidation of THF inhibited by hydroxyl TEMPO (**2**), experimental trace (red) and simulated trace (green). Y-axis: [O_2].

References

S1 Cicco S. R.; Vona D.; De Giglio E.; Cometa S.; Mattioli-Belmonte M.; Palumbo F.; Ragni R.; Farinola G. M. Chemically Modified Diatoms Biosilica for Bone Cell Growth with Combined Drug-Delivery and Antioxidant Properties. *Chem. Plus. Chem* **2015**, *80*, 1104 –1112.

S2 Maillard B.; Ingold K. U.; Scaiano J. C. Rate constants for the reactions of free radicals with oxygen in solution. *J. Am. Chem. Soc.* **1983**, *105*, 5095-5099

S3 Amorati R.; Baschieri A.; Morroni G.; Gambino R.; Valgimigli L. Peroxyl Radical Reactions in Water Solution: A Gym for Proton-Coupled Electron-Transfer Theories. *Chem. Eur. J.* **2016**, *22*, 7924 – 7934
